# Expression of GATA3 in MDA-MB-231 Triple-negative Breast Cancer Cells Induces a Growth Inhibitory Response to TGFß

**DOI:** 10.1371/journal.pone.0061125

**Published:** 2013-04-08

**Authors:** Isabel M. Chu, Wei-Chu Lai, Olga Aprelikova, Lara H. El Touny, Hosein Kouros-Mehr, Jeffrey E. Green

**Affiliations:** Transgenic Oncogenesis and Genomics Section, Laboratory of Cancer Biology and Genetics, Center for Cancer Research, National Cancer Institute, National Institutes of Health, Bethesda, Maryland, United States of America; AMS Biotechnology, United Kingdom

## Abstract

Transforming growth factor (ß1TGFß1) can promote proliferation in late stage cancers but acts as a tumor suppressor in normal epithelial cells and in early stage cancers. Although, the TGFß pathway has been shown to play a key role in tumorigenesis and metastasis, only a limited number of models have been developed to understand this process. Here, we present a novel model system to discern this paradoxical role of TGFß1 using the MDA-MB-231 (MB-231) cell line. The MB-231 triple-negative breast cancer cell line has been extensively characterized and has been shown to continue to proliferate and undergo epithelial-to-mesenchymal transition (EMT) upon TGFß1 stimulation. We have previously shown by microarray analysis that expression of GATA3 in MB-231 cells results in reprogramming of these cells from a basal to a luminal subtype associated with a reduction of metastasis and tumorigenesis when implanted as xenografts. We now demonstrate that GATA3 overexpression in these cells results in a reduction of TGFß1 response, reversal of EMT, and most importantly, restoration of sensitivity to the inhibitory effects on proliferation of TGFß1. Microarray analysis revealed that TGFß1 treatment resulted in reduction of several cell cycle effectors in 231-GATA3 cells but not in control cells. Furthermore, our microarray analysis revealed a significant increase of BMP5 in 231-GATA3 cells. We demonstrate that combined treatment of MB-231 control cells with TGFß1 and BMP5 results in a significant reduction of cellular proliferation. Thus, this model offers a means to further investigate potentially novel mechanisms involved in the switch in response to TGFß1 from tumor promoter to tumor suppressor through the reprogramming of a triple-negative breast cancer cell line by the GATA3 transcription factor.

## Introduction

GATA3 is a transcription factor belonging to the GATA family of Zn-finger family members. GATA3 has been mainly implicated in cell fate decisions during development and differentiation of the hematopoietic cell lineages [1] and more recently, of mammary gland development [2], [3]. GATA3 is critical for luminal differentiation during mammary gland development and is expressed only in the ducts and terminal end buds (TEB) of luminal cells [2]. Loss of GATA3 expression has been associated with a worse prognosis in breast cancer patients [4]. Our lab and others have shown that overexpression of GATA3 in the metastatic MDA-MB-231 (MB-231) basal triple-negative breast cancer cell line reduces tumorigenesis and metastasis [5]–[7]. Here we show that GATA3 promotes a mesenchymal-to-epithelial transition (MET) in MB-231 cells, reduces TGFß dependent epithelial-to-mesenchymal transition (EMT) response and most importantly, results in a TGFß cytostatic effect in the metastatic cell line, MB-231.

EMT is a reversible process that involves loss of an epithelial phenotype and a concomitant acquisition of a mesenchymal phenotype. EMT is present during embryogenesis and tissue development and is often recapitulated during tumor progression, resulting in increased invasiveness and a more aggressive phenotype [8], [9]. EMT is characterized by loss of apical-basolateral cell polarity, actin reorganization and increased extracellular matrix protein deposition resulting in increased migration and invasion [10]. One of the hallmarks of EMT is the downregulation or loss of E-cadherin [9]. E-cadherin is transcriptionally repressed by ZEB1, ZEB2, SNAI1, SNAI2, Twist1, Twist2 and E12/E47 [11]. E-cadherin loss promotes metastasis through induction of EMT, invasiveness and anoikis resistance [12]. Cancer cells undergo localized EMT at the invasive front of the tumor and extracellular cues, including activation of TGFß and Wnt at the tumor front, and expression of EMT markers prime cells for metastatic dissemination [13].

The role of the pleiotroic cytokine transforming growth factor ß1 (TGFß1), a potent inducer of EMT and tumor progression in many types of cancers including breast cancer, has been well documented [14]. TGFß1 belongs to the TGFß superfamily and has been implicated in regulating proliferation, differentiation, adhesion, apoptosis, migration, homeostasis and tissue repair [15]. Binding of TGFß to the TGFß type II receptor (TGFßRII) leads to receptor activation, heterodimerization and phosphorylation of the TGFß type I receptor (TGFßRI) at a glycine-serine rich domain. The TGFßRI can then recruit, phosphorylate and activate the receptor-regulated Smads - Smad2 and Smad3 (R-Smads) - whereby phosphorylated Smad2/3 accumulate in the nucleus and bind to the common partner Smad 4 (co-Smad). These Smad complexes regulate transcriptional activators or repressors of gene expression.

Although TGFß response is growth inhibitory in most epithelial cells, advanced tumors of epithelial origin often show oncogenic responses to TGFß [16]. During mammary gland development, TGFß plays a mostly growth inhibitory role in mammary epithelial cells and is involved in branching morphogenesis, lactation and involution [17]. In breast cancer, TGFß acts as a tumor suppressor during early stages of cancer development. In contrast, TGFß acts as a tumor promoter at later stages of tumorigenesis and promotes metastatic spread [16], [18].

This paradoxical role of TGFß in breast cancer has been widely studied in different models of breast cancer. Tang and colleagues investigated TGFß response using a panel of MCF10A-derived cell lines that included the weakly tumorigenic MCF10Ca1h line (designated MIII in this manuscript) which retained tumor suppressor responses to TGFß and the metastatic, highly tumorigenic MCF10Ca1a.cl1 line in which tumor suppressor responses to TGFß were lost and pro-metastatic responses were unmasked [19]. In MMTV-Neu-induced mammary tumorigenesis, activation of the TGFß signaling pathway through the use of an activated TGFßRI resulted in increased lung metastasis, but increased the latency of primary tumor formation, whereas a dominant-negative TGFßRII resulted in the converse effects [20]. Although the existing models to investigate TGFß1 have shed some light into the mechanisms involved in pro-oncogenic vs. tumor suppressive functions, there are some limitations to the established models for studying the effects of TGFßExisting cell lines used to investigate the paradoxical role of TGFß are either derived from different cellular origins or have been manipulated through passage in mice where the cells may undergo additional modifications.

Here we present a novel isogenic cell model system in which only one gene, GATA3, was ectopically expressed in MB-231 cells that dramatically alters the cellular response to TGFßUsing this model system, we have shown that GATA3 alone is sufficient to resensitize this cell line to the cytostatic effects of TGFß and provide a putative mechanism for this change in response to TGFßthrough alterations in the expression of cell cycle regulators and genes that regulate EMT. This model system provides new insights into how a differentiation transcription factor, GATA3, is involved in regulating the TGFß response.

## Materials and Methods

### Cell lines, transfection and lentivirus infection

MB-231 cells were obtained from American Type Culture Collection (ATCC) and maintained in DMEM/high glucose media (Life Technologies, Grand Island, NY) supplemented with 10% fetal bovine serum (FBS) (Life Technologies), penicillin/streptomycin (Life Technologies) and sodium pyruvate (Life Technologies). For TGFß1 treatment, cells were grown in DMEM/high glucose supplemented with serum replacement (SR, Sigma, St. Louis, MO) for 24 hrs prior to TGFß1 stimulation. Cells were treated with 2 ng/ml of TGFß. MB-231 cells stably expressing green fluorescent protein (GFP) or the GATA3 transcription factor and GFP were made as previously described [5]. MCF10Ca1h [21] cells were kindly provided by Lalage Wakefield, NCI.

### Construction of E-cadherin (CDH11) reporter construct

The E-cadherin reporter construct was generated using the 4 kb promoter fragment upstream of the E-cadherin first exon that was PCR amplified from a BAC genomic DNA template (human BAC 354M1) using the following primers: Forward GAGGTACCTGATACAAGAGAAAA CAGAGGGC and Reverse GAGACGCGTGGCTGGCCGGGGACGCCGAG. The promoter fragment was then cloned into the KPN1 and Mlu1 sites of the PGL3-luciferase reporter plasmid (Promega, Madison, WI).

### Luciferase activity

For luciferase activity, 231-Empty and 231-GATA3 cells were seeded on 24 well plates, transfected for 24 hrs with E-cadherin reporter construct and renilla luciferase, samples were collected and luciferase activity measured using Wallac Victor^2^ (Perkin Elmer, Waltham, MA). Samples were normalized to renilla activity.

### Methylation-specific PCR

Cells were treated with vehicle or 5-Aza-2′-deoxycytidine (5-AZA, Sigma) for 4 days prior to DNA isolation. DNA was isolated from stably transduced 231-Empty and 231-GATA3 cells using the DNeasy Blood and Tissue Kit (Qiagen, Valencia, CA) according to the manufacturer's protocol. DNA was sulfonated using the EZ DNA Methylation™ Kit (ZYMO Research, Irvine, CA). Methylation-specific PCR was performed with the following primer sets: 5′- GGTGAATT TTTAGTTAATTAGCGGTAC -3′ and 5′- CATAACTAACCGAAAACGCCG-3′ for methylated CDH1; 5′- GGTAGGTGAATTTTTAGTTAATTAGTGGTA-3′ and 5′- ACCCATAACTAACC AAAAACACCA-3′ for unmethylated CDH1 (211 bp) [22]. Bisulfite-treated DNA was PCR-amplified with annealing temperatures of 57°C for both methylated and unmethylated CDH1 DNA.

### Quantitative reverse transcription PCR (Q-RT-PCR)

cDNA was synthesized from 1 µg of total RNA using the Superscript III kit (Life Technologies). Q-RT-PCR was performed using IQ SYBR Green Supermix (Bio-Rad Laboratories, Hercules, CA) and an iCycler Thermal Cycler (Bio-Rad Laboratories). The quantity of mRNA was normalized to the housekeeping gene cyclophilin B.

### Immunoblotting and antibodies

Cells were lysed in ice-cold RIPA buffer for Western blot analyses as described previously [5]. GATA3 (558686) and p21 (sc-397) antibodies were purchased from Santa Cruz (Santa Cruz, CA); Twist (ab50887),ß-catenin (ab32572), Smad3 (ab28379) were from Abcam (Cambridge, MA); Snail (3895), Phospho-Smad2 (3108), Phospho-Akt (Ser473, 9271), Akt (9272), p44/42 MAP Kinase (9102), Phospho-p44/42 MAPK (9106), E-cadherin (4065), Smad2 (3103), Phospho-Smad1/5/8 (9511) and Slug (9585) were purchased from Cell Signaling Technology (Danvers, MA); Phospho-Smad3 (1880-1) was purchased from Epitomics (Burlingame, CA); Smad1 (385400) was purchased from Life Technologies.

### Knock down of GATA3 or BMP5

3×10^5^ cells in 6-well plates were transfected with 60 nM siRNA for GATA3 (Dharmacon, J-003781-07 and J-003781-09) or siRNA for BMP5 (Qiagen, S100129094 and S100129108) or non-targeting control using Oligofectamine Reagent (Invitrogen) according to the manufacturer's protocol. To knock down GATA3 in 231-GATA3 cells, transfection with siRNA was performed on two consecutive days. On the third day, cells were placed in serum–free media followed by TGFß treatment as above. After 24 hours cells were labeled with BrdU and subjected to flow cytometry analysis.

### Microarray data processing

Total RNA was isolated using Trizol® according to the manufacturer's protocol (Life Technologies). RNA quality was checked using an Agilent Bioanalyzer (Agilent, Santa Clara, CA, USA). All samples used for microarray analysis had a high quality score (RIN>9). RNA (1 µg) was reverse transcribed with a T7-oligo(dT) primer and biotin labeled using the Affymetrix One Cycle Target Labeling kit (Affymetrix, Santa Clara, CA) following the manufacturer's protocol. For 231-Empty vs. 231-GATA3 cells, samples were analyzed using BRB-ArrayTools developed by Richard Simon and the BRB-ArrayTools Development Team (http://linus.nci.nih.gov/BRB-ArrayTools.html) as previously described [5]. For 231-Empty or 231-GATA3 cells in serum replacement (SR) −/+ transforming growth factor beta-1 (TGFß1, four replicates of each group were prepared, labeled, and hybridized to Affymetrix Human Gene 1.0 ST array GeneChips and scanned on an Affymetrix GeneChip Scanner 3000. Data were collected using Affymetrix GCOS software and processed into log base 2 gene expression measures using the RMA algorithm and quantile normalization using PARTEK software (PARTEK, St. Louis, MO). Network analysis was done using Ingenuity Pathway Analysis (Redwood City, CA).

### Flow cytometry

Cell cycle profiles were assayed by 5-bromo-2-deoxyuridine (BrdU) pulse labeling and flow cytometric analysis was performed as previously described [5] using FACScalibur (Becton Dickinson, Franklin Lakes, NJ).

### Statistical analyses

Q-RT-PCR mean differences were tested with an unpaired Student's *t*-test. Statistical significance was depicted in the figures as *P<0.05, **p<0.01 and ***p<0.001. All *in vitro*, experiments were repeated at least 3 times. Differentially expressed genes between 231-Empty and 231-GATA3 cells grown in full serum were identified with univariate unpaired Student's t-test and p-value threshold of 0.001, using the BRB-ArrayTools statistical software. Differentially expressed genes between 231-Empty and 231-GATA3 cells grown in SR −/+ TGFßwere identified using PARTEK software.

## Results

### GATA3 promotes expression of epithelial markers and loss of mesenchymal markers in MB-231 cells

**Figure 1:**
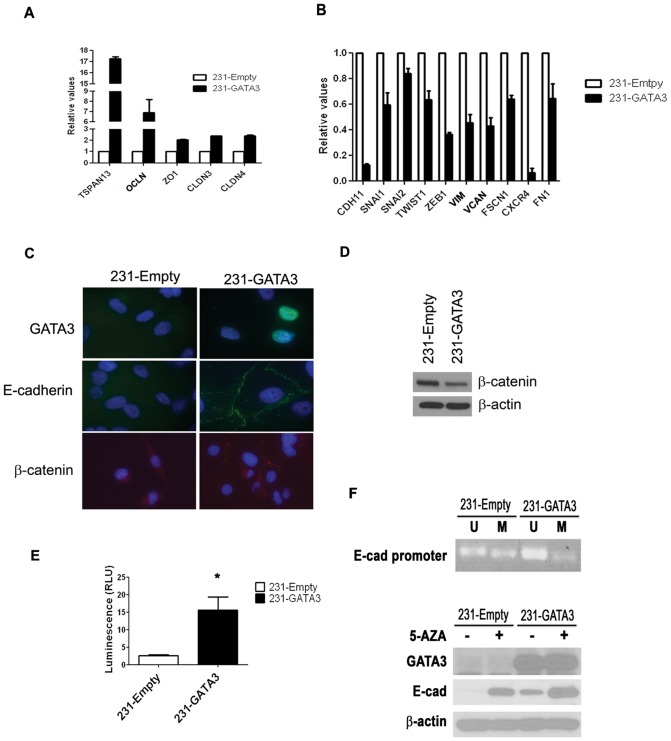
GATA3 over-expression in MB-231 cells promotes EMT and the re-expression of E-cadherin. (A) Q-RT-PCR demonstrates increased expression of epithelial markers in 231-GATA3 cells compared to 231-Empty cells. We observed increased TSPAN13, OCLN, ZO1, CLDN3 and CLDN4 in 231-GATA3 vs. 231-Empty cells. Samples were normalized to cyclophilin B. (B) Q-RT-PCR showing that the relative expression of mesenchymal and metastatic markers is reduced in 231-GATA3 cells compared to 231-Empty cells. We observed reduced CDH1, SNAI1, SNAI2, TWIST1, ZEB1, VIM, VCAN, FSCN1, CXCR4 and FN1 in 231-GATA3 vs. 231-Empty cells. Samples were normalized to cyclophilin B. (C) Immunofluorescence of 231-Empty and 231-GATA3 cells for GATA3, E-cadherin and ß-catenin. GATA3 over-expression in MB-231 results in re-expression of E-cadherin associated with the cell membrane and re-distribution of ß-catenin. (D) Western blot showing reduced expression of ß-catenin in 231-GATA3 cells compared to 231-Empty cells. ß-actin was used as loading control. (E) Luciferase activity showing increased activity of the E-cadherin reporter construct in 231-GATA3 cells vs. 231-Empty cells. (F) top panel: PCR using primers that detect only unmethylated or methylated DNA at the E-cadherin promoter of 231-Empty and 231-GATA3 cells. DNA underwent bisulfate treatment prior to the PCR reaction. 231-GATA3 cells show reduced methylated and increased unmethylated DNA in the E-cadherin promoter region compared to 231-Empty cells. Lower panel: western blot of 231-Empty and 231-GATA3 cells for E-cadherin. Cells were treated with 5-AZA for 4 days prior to lysate collection. Treatment of 231-Empty and 231-GATA3 cells with 5-AZA increased E-cadherin expression.

The derivation of 231-Empty and 231-GATA3 cells has been previously described [5]. Our lab and others have demonstrated that the overexpression of GATA3 in MB-231 cells results in morphological changes from a spindloid phenotype to a cuboidal, epithelial appearance in 2D culture, a more organized structure in 3D culture, and a significant reduction in tumorigenesis and metastasis when implanted as xenografts [5]–[7]. In addition to the morphological and biological changes induced by GATA3 in MB-231 cells that we described previously [5], our analyses of microarray data comparing 231-Empty cells with 231-GATA3 cells demonstrated a significant reduction in gene expression associated with EMT and an increase in expression of genes associated with MET. Q-RT-PCR analysis confirmed an increase in expression of Tetraspanin 13 (TSPN13), Occludin (OCLN), Zona occludens-1 (ZO1), Claudin 3 (CLDN3) and Claudin 4 (CLN4) (Figure 1A), which are genes associated with an epithelial or less aggressive phenotype. In contrast, the expression of genes associated with a mesenchymal phenotype were reduced including Cadherin-11 (CDH11), Snail1 (SNAI1), Slug (SNAI2), Twist (TWIST1), Zinc finger E-box-binding homeobox 1 (ZEB1), Vimentin (VIM), Versican (VCAN), Fascin homolog 1 (FSCN1), C-X-C chemokine receptor type 4 (CXCR4) and Fibronectin 1 (FN1) (Figure 1B).

Loss of E-cadherin expression is considered a hallmark of EMT [9]. MB-231 cells lack E-cadherin expression and exhibit a mesenchymal phenotype [23]. Immunofluorescence for E-cadherin and GATA3 protein revealed expression of E-cadherin in the cytoplasmic membrane only in 231-GATA3 cells, but no detectable expression in control 231-Empty cells (Figure 1C). In epithelial cells, ß-catenin is co-localized with E-cadherin on the cellular membrane. Immunofluorescence staining for ß-catenin revealed homogenous diffuse staining in 231-Empty cells while 231-GATA3 cells showed relocalization of ß-catenin to the cellular membrane consistent with E-cadherin expression and localization (Figure 1C). Furthermore, western blot analysis also revealed reduction of ß-catenin in 231-GATA3 cells compared to 231-Empty cells (Figure 1D). We generated a luciferase reporter construct containing the 4 kb 5′ genomic promoter region of E-cadherin and observed a 6-fold increase in luciferase activity when this construct was transfected into 231-GATA3 cells compared to 231-Empty cells (p<0.05, Figure 1E) suggesting that GATA3 can upregulate E-cadherin expression at the transcriptional level.

E-cadherin has previously been shown to be regulated by methylation in MB-231 cells [23]. Therefore, we investigated the methylation status of E-cadherin in 231-GATA3 and 231-Empty cells. By Methylation Specific PCR (MSP), we observed a reduction of methylated DNA at the E-cadherin promoter in 231-GATA3 cells compared to 231-Empty cells, whereas unmethylated DNA was reduced in 231-Empty vs. 231-GATA3 cells (Figure 1F). These changes in promoter methylation status correspond to the total E-cadherin protein levels as determined by western blot (Figure 1F). 231-GATA3 cells re-expressed E-cadherin protein, whereas E-cadherin protein was undetectable in 231-Empty cells by western blot. Treatment with 5-Aza-2′-deoxycytidine (5-AZA), a demethylating agent, resulted in detectable E-cadherin protein expression in 231-Empty cells (Figure 1F). 5-AZA also increased expression of E-cadherin in 231-GATA3 cells. These findings suggest that there are methylated sites in the E-cadherin promoter that are independent of GATA3 expression in addition to the GATA3 dependent methylated sites.

### GATA3 overexpression promotes changes in the TGFß signaling pathway

**Figure 2:**
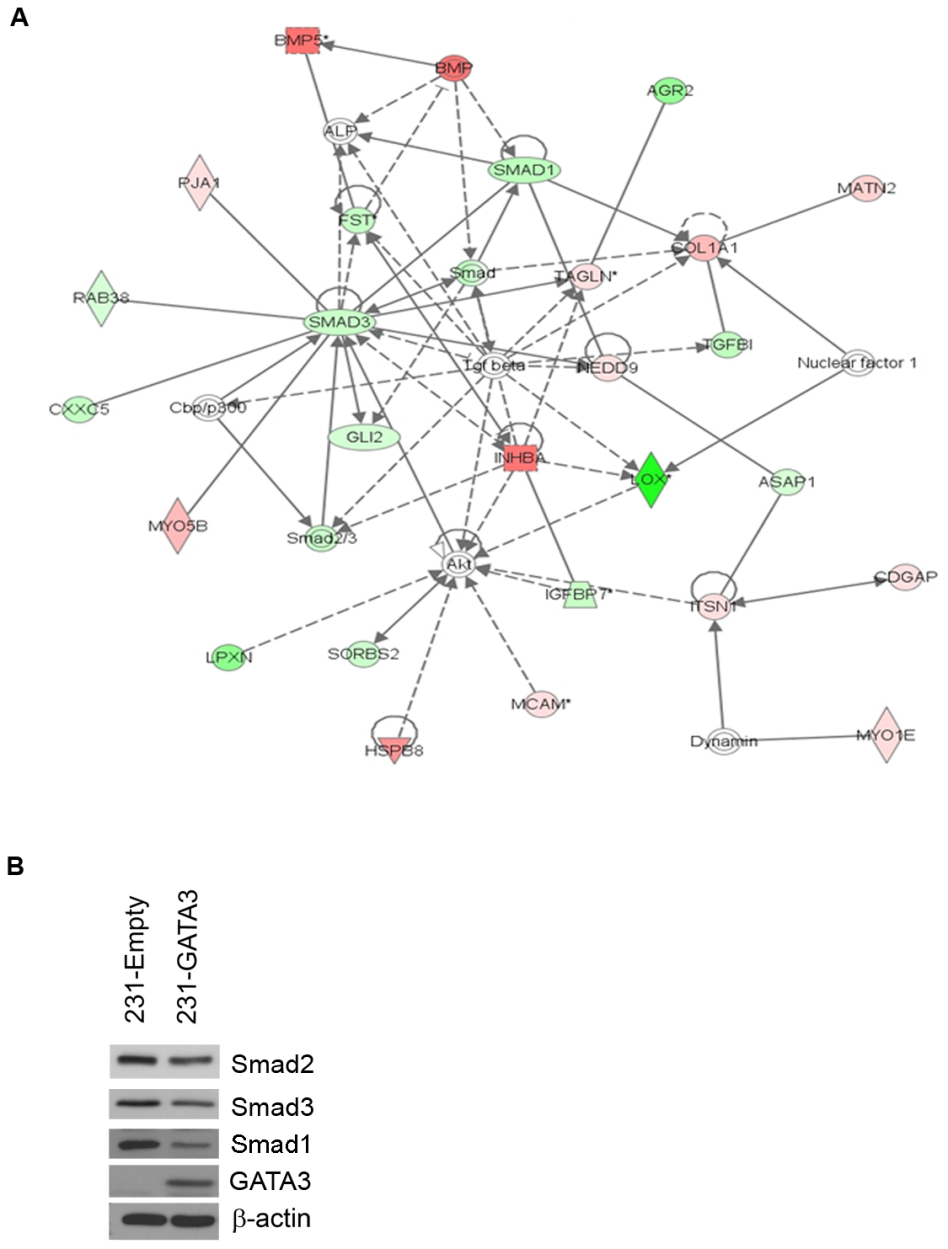
GATA3 reduces effectors in the TGFß1 pathway in MB-231 cells. (A) Ingenuity network analysis of 231-Empty vs. 231-GATA3 cells. Genes that were differentially expressed in 231-Empty vs. 231-GATA3 cells (p<0.001, fold change >1.5) were input into Ingenuity Pathway analysis. One of the top networks identified demonstrated changes in several TGFß1 effectors. (B) Western blot of 231-Empty and 231-GATA3 cells validating changes in expression of Smad2, Smad3 and Smad1 between 231-Emtpy and 231-GATA3 cells.

We have previously performed Affymetrix microarray analysis of 231-Emtpy and 231-GATA3 cells to further discern the putative mechanisms utilized by GATA3 to promote changes in EMT. Probes were selected with a 1.5 fold change in expression and based on a nominal 0.001 level of the univariate test. Of these 872 probes, 832 probes were mapped to 619 unique genes using Ingenuity Pathway Analysis. Ingenuity network analysis revealed that one of the top networks altered upon GATA3 over-expression in MB-231cells were genes involved in development and morphology (Figure 2A). This network showed a reduction in the expression of effectors involved in the TGFß signaling pathway (SMAD1, 2 and 3) in 231-GATA3 vs. 231-Empty cells (Figure 2A). Western blot analysis confirmed a reduction of several of the TGFß effectors including Smad1, 2 and 3 in 231-GATA3 cells compared to 231-Empty cells (Figure 2B).

### GATA3 overexpression reduces the transcriptional response to TGFßand TGFß-dependent EMT stimulation of MB-231 cells

**Figure 3:**
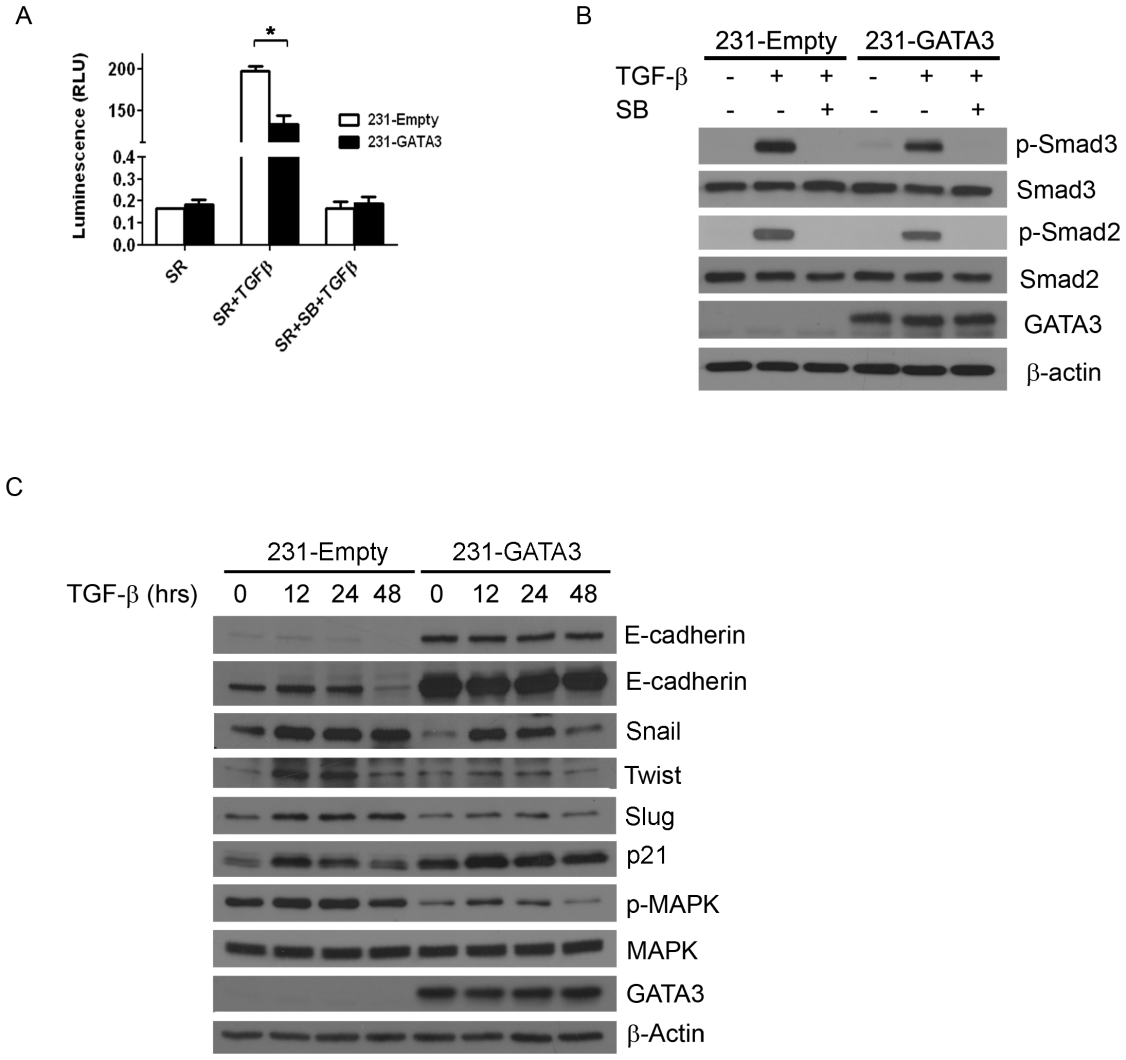
GATA3 reduces the TGFß1 transcriptional response in MB-231 cells and abrogates TGFß1 mediated EMT. (A) Luciferase activity of 231-Empty and 231-GATA3 cells using the TGFß response reporter construct CAGA12. 231-GATA3 cells exhibited a reduced TGF-ß1-dependent activation of CAGA12 compared to 231-Empty cells. (B) Western blot analysis of 231-Empty and 231-GATA3 cells showing changes in activation of SMAD signaling in response to TGF-ß1. 231-GATA3 cells treated with TGF-ß1 for 1 hr. showed reduced p-SMAD 3 and p-SMAD 2 levels compared to 231-Empty cells. ß-actin was used as loading control. (C) Western blot analysis showing differences in expression of EMT markers at the indicated times. Cells were grown in SR media and treated with TGF-ß1 for 12, 24 or 48 hrs.

TGFß has been shown to stimulate EMT [14]. Since 231-GATA3 cells showed reduced expression of EMT markers suggesting a transition towards an epithelial phenotype, we investigated whether the observed MET phenotypic changes were associated with a reduction in response to TGFß-signaling. To investigate this, 231-Empty and 231-GATA3 cells were transfected with the TGFß-dependent CAGA12-luciferase reporter construct, grown in the absence of TGFß1 for 24 hours under serum replacement (SR) conditions, treated with TGFß1 for 16 hrs and harvested for luciferase activity measurements. Basal luciferase activity was observed in SR media in both 231-Empty and 231-GATA3 cells. Upon TGFß1 stimulation, reporter activity in 231-Empty cells was significantly increased compared to SR only treated cells, whereas in cells expressing GATA3, reporter activity was reduced by 33% (Figure 3A; p<0.05). Pretreatment of cells with the TGFß1 inhibitor, SB431542, prior to TGFß1 stimulation abrogated activation of the luciferase reporter (Figure 3A). Western blot analysis revealed reduced phosphorylated Smad 2 and 3 following 1 hr treatment with TGFß1 in 231-GATA3 cells compared to 231-Empty cells (Figure 3B). Therefore the reduced phosphorylated Smad2/3 levels may account for the observed reduced luciferase activity in 231-GATA3 cells compared to 231-Empty cells.

To determine whether GATA3 alters the TGFß-dependent EMT response in MB-231 cells, we investigated the ability of TGFß1 to induce EMT markers in 231-Emtpy and 231-GATA3 cells. The cells were treated with TGFß1 for 12, 24 or 48 hrs and expression of several EMT markers was analyzed (Figure 3C). Upon TGFß1 treatment, a more than 50% reduction in E-cadherin expression was observed by 48 hrs compared to control SR-conditions for the 231-Empty cells, whereas for 231-GATAT3 cells, E-cadherin levels remained very similar to control SR-conditions following 48 hrs of TGFß1 stimulation (Figure 3C). The reduction of E-cadherin protein by TGFß1 in 231-Empty cells compared to 231-GATA3 cells was more readily observed with a longer exposure time of the western blot (Figure 3C). TGFß1 stimulation of 231-Empty cells resulted in a 2.1-fold induction of Snail within the first 12 hrs that persisted for 48 hrs (Figure 3C). However, 231-GATA3 cells exhibited reduced levels of Snail under SR conditions (0.4 fold compared to control 231-Empty cells). The induction of Snail did not persist as it did in the 231-Empty cells since by 48 hrs expression levels were reduced and approached levels similar to SR-only conditions. Induction of Twist upon TGFß stimulation was marginal with a 1.6 fold induction at 12 and 24 hrs in 231-GATA3 cells compared to SR conditions, whereas 231-Empty cells showed a 5.5 fold induction at 12 and 24 hrs compared to SR conditions. By 48 hrs, Twist expression in 231-Empty control cells still showed a 2-fold increase compared to SR alone whereas 231-GATA3 cells showed a 2-fold reduction compared to SR alone. Similar findings were observed for expression of Slug in response to TGFß1. Slug was induced 1.9 fold compared to SR alone in 231-Empty cells which persisted for 48 hrs, whereas 231-GATA3 cells showed a more transient induction of Slug that did not persist by 48 hrs. Similarly, we observed reduced p-MAPK in 231-GATA3 cells compared to 231-Empty cells under SR-conditions or upon TGFß1 stimulation (Figure 3C). In contrast, p21 showed opposite effects in response to TGFß1 with increased p21 expression in 231-GATA3 cells compared to 231-Empty cells under SR-conditions and upon TGFß1 stimulation (Figure 3C).

### GATA3 inhibits the TGFß proliferative response in MB-231 cells

**Figure 4:**
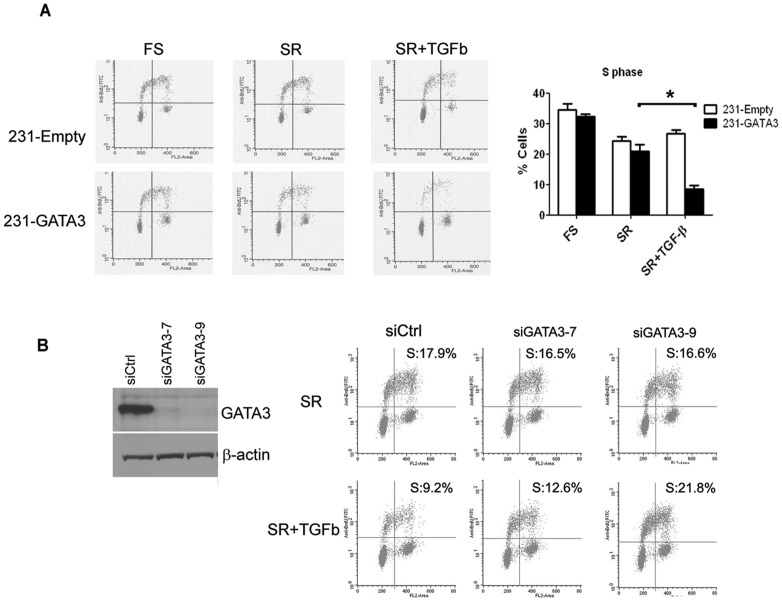
GATA3 confers growth inhibitory response to TGFß1 in MB-231 cells. (A) Flow cytometric analysis of 231-Empty and 231-GATA3 cells grown in full serum, SR (serum replacement) or SR +TGFß for 24 hrs. 231-GATA3 cells show a reduction in %S-phase cells upon TGF-ß1 treatment compared to SR conditions whereas 231-Empty cells continue to proliferate with TGF-ß1 treatment. (B) Western blot analysis of GATA3 downregulation by siRNA and flow cytometric analysis of TGFb effect in control or GATA-3 siRNA transfected cells. Percentage of cells in S-phase is provided on each panel.

The response of breast cancers to TGFßhas been shown to be contextual depending on the stage of disease. Early stage breast cancers or less invasive breast cancer cells are sensitive to the cytostatic effects of TGFß1compared to their more aggressive counterparts that may acquire growth and proliferative advantages in response to TGFß [16], [19]. Since GATA3 reduces tumorigenesis and metastatic potential of MB-231 cells [5], we investigated the proliferative response of 231-Empty and 231-GATA3 cells to TGFß1. Both 231-Empty and 231-GATA3 cells have similar basal proliferation rates as measured by % S phase in full serum or in SR conditions where TGFß1 is absent (Figure 4A). The rate of proliferation for 231-Empty cells was not inhibited upon 24 hr stimulation with TGF-ß1 (Figure 4A). However, an inhibitory response to TGFß1 was observed in 231-GATA3 cells (Figure 4A). Percent S-phase of 231-Empty cells did not show a statistical significant change when cells in SR-conditions were stimulated with TGFß1 (24% and 27% in S-phase, respectively). However, % S-phase of 231-GATA3 cells grown under SR-conditions was remarkably reduced upon TGFß1 stimulation (21% vs. 8% in S-phase, respectively, p<0.05). To further demonstrate that this inhibitory effect on proliferation by TGFß1 is mediated by GATA3 we knocked down GATA3 expression in 231-GATA3 cells (Figure 4B). Treatment with TGFß1 showed robust suppression of S phase in 231-GATA3 cells transfected with non-targeting control, but knock down of GATA3 with two different siRNAs diminished or completely reversed the suppressive effect on proliferation in response to TGFß (Figure 4B).

### GATA3 alters the gene expression profile of MB-231 cells in response to TGFß1

**Figure 5:**
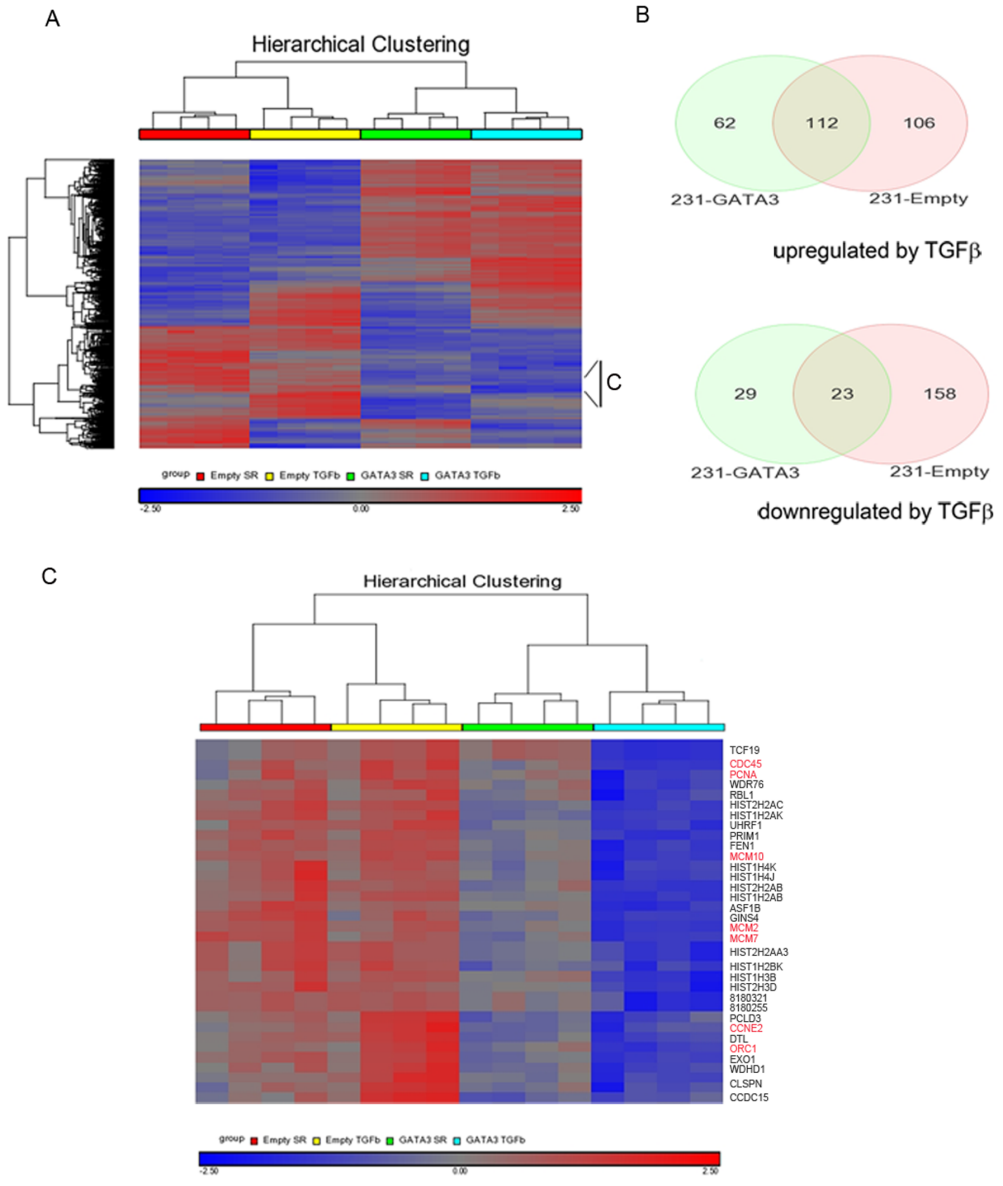
Microarray analysis of 231-Empty and 231-GATA3 cells in response to TGF-ß1. (A) Unsupervised hierarchical clustering analysis of 231-Empty and 231-GATA3 cells with or without TGFß1 treatment for 24 hrs. The displayed expression of each gene was standardized within the respective datasets (gene-z-score transformation for the 4 treatment groups). The black bar shows genes in panel C below. (B)Venn diagram of genes that show >1.6 fold difference and p<0.001 in response to TGFß1 in 231-Empty and 231-GATA3 cells. (C) Expanded cluster from indicated region in (A) showing changes in genes associated with cell cycle regulation.

**Figure 6:**
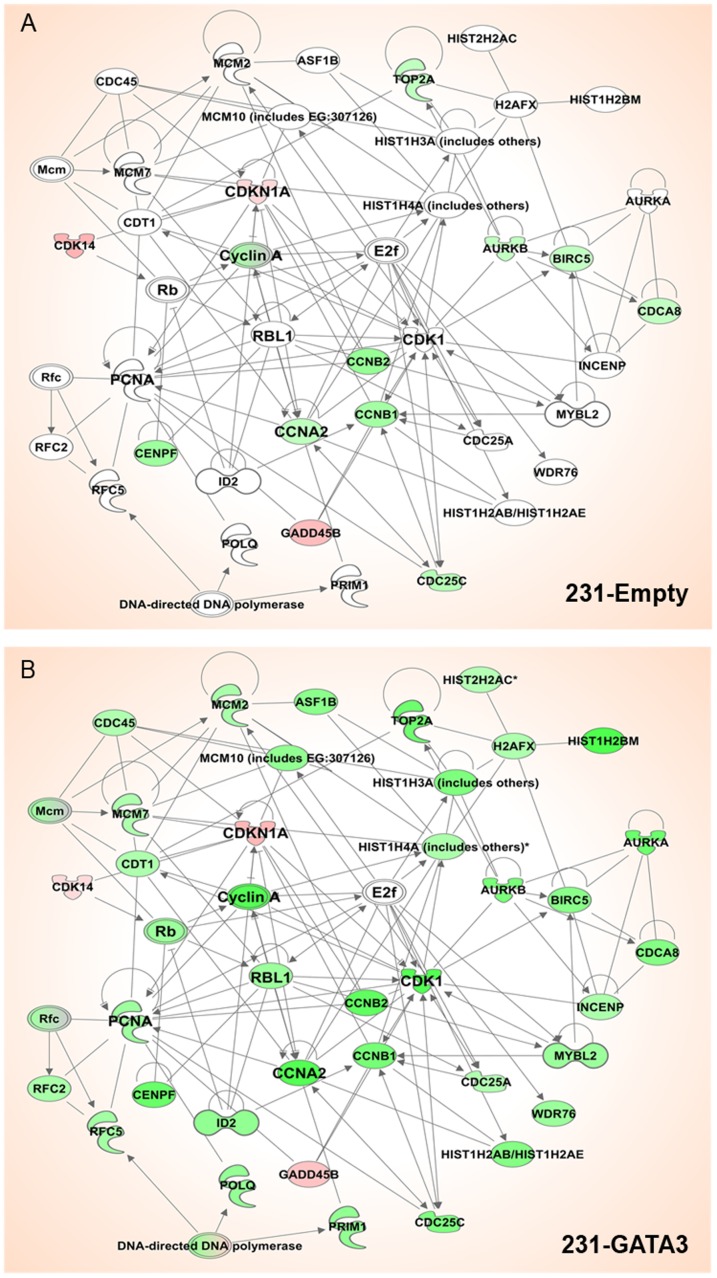
Network of genes involved in cell cycle regulation. Network of genes that were differentially expressed between 231-Empty and 231-GATA3 by >1.5 fold and p<0.001 (Ingenuity Pathway Analysis). Green represents reduced gene expression upon TGFß1 stimulation compared to untreated cells and red represent increased gene expression compared to untreated cells. Intensity of the color represents the degree of change for (A) 231-Emtpy cells and (B) 231-GATA3 cells. Many more genes in this network are downregulated by TGFß1in 231-GATA3 cells compared to 231-Empty cells.

The expression of GATA3 in MB-231 cells resulted in the cells becoming growth inhibited in response to TGFß1compared to 231-Empty cells. In order to identify GATA3-dependent transcriptional changes associated with this change in response to TGFß, we performed microarray analysis of 231-Empty and 231-GATA3 cells cultured in SR media and stimulated with TGFß1 for 24 hrs. Pairwise comparisons using PARTEK analysis software was performed between the cells cultured under the following conditions: 231-Empty cells SR vs. 231-Empty cells SR+ TGFß; 231-GATA3 SR vs. 231-GATA3 SR+ TGFß; 231-Emtpy SR vs. 231-GATA3 SR; and 231-Empty + TGFß vs. 231-GATA3+TGFß. A total of 1252 genes were found to be differentially expressed between these treatment groups (>1.6 fold change at p<0.001). The heatmap of the 1252 genes across the four cell treatment groups using unsupervised hierarchical clustering analysis demonstrated distinctions in gene expression among the four groups (upregulated (red) and downregulated (blue) regions) (Figure 5A). Of the 218 genes upregulated in 231-Empty cells upon TGFß1 treatment, only 112 genes were also upregulated in 231-GATA3 cells (Figure 5B). Furthermore, we observed 62 genes that were only upregulated in 231-GATA3 cells in response to TGFß1. Similarly, of the 181 genes downregulated in response to TGFß1 in 231-Empty cells, only 23 genes were also downregulated in 231-GATA3 cells, whereas 29 genes were downregulated only in 231-GATA3 cells (Figure 5B). Further analysis of the set of genes differentially regulated in 231-GATA3 and 231-Empty cells in response to TGFß1 revealed a cluster that contained genes primarily involved in cell proliferation including PCNA, ORC1, CDC45, CCNE2, MCM2 and 7 (Figure 5A and 5C). Genes in this cluster showed either no change or increased expression in response to TGFß1 in 231-Empty cells, whereas TGFß1 stimulation of 231-GATA3 cells reduced the expression of these genes (Figure 5C). The acquired response of 231-GATA3 cells to the reduced expression of proliferative genes in response to TGFß1 may account for the switch from a proliferative to growth inhibitory response of the 231-GATA3 cells. To further explore the differences in TGFß1 response between 231-Empty vs. 231-GATA3 cells, we performed a network analysis using Ingenuity Pathway Analysis and compared changes in the expression of genes associated with cell cycle functions upon TGFß stimulation in 231-Empty and 231-GATA3 cells (Figure 6). This network analysis demonstrated changes in the expression of genes associated with the cell cycle (green indicates a reduction in gene expression upon TGFß treatment, red indicates increased expression, and the intensity of the color represents the degree of change). Among some of the genes that were only reduced or showed a greater degree of reduction in 231-GATA3 vs. 231-Empty cells were Cyclin A, CDK1 and PCNA. Interestingly, we also observed a greater increase in p21 expression in 231-GATA3 vs. 231-Empty in response to TGFß1. These observed changes in cell cycle effectors likely account for the observed restoration to the cytostatic effects of TGFß1 in 231-GATA3 vs. 231-Empty cells.

### BMP5 treatment of 231-Empty cells restores TGFßresponse

**Figure 7:**
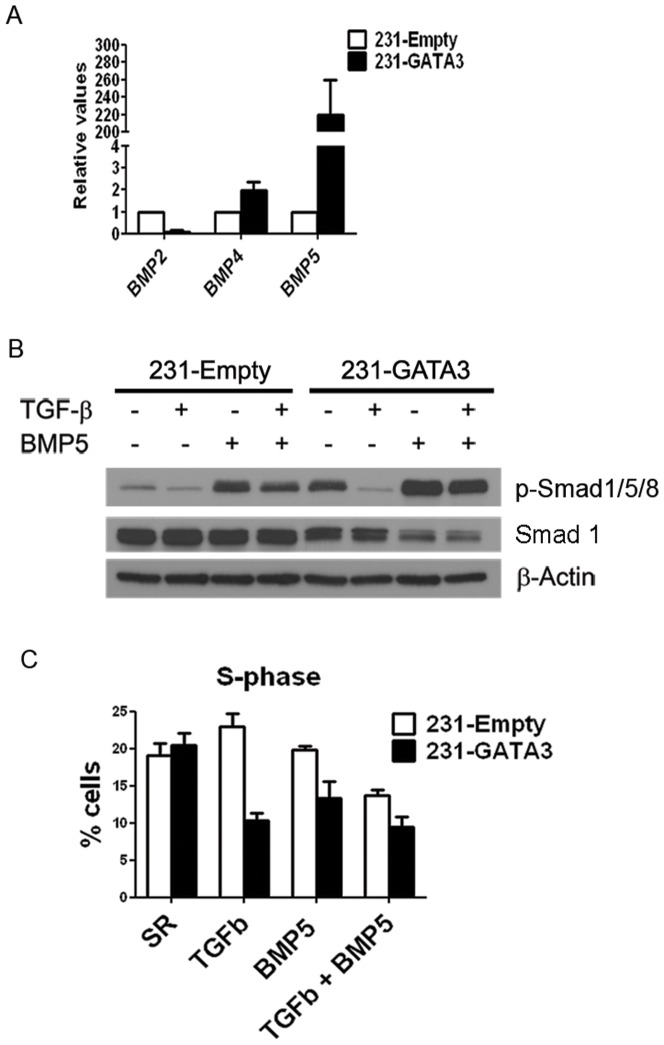
GATA3 upregulates BMP5 in MB-231 cells. (A) Q-RT-PCR showing expression of BMP2, BMP4 and BMP5 in 231-Empty and 231-GATA3 cells. GATA3 significantly upregulates BMP5 expression in MB-231 cells. (B) Western Blot analysis showing increased pSMAD1/5/8 in TGFß1+BMP5 treated 231-GATA3 cells compared to 231-Empty cells. ß-actin was used as a loading control. (C) BrdU incorporation in pulse-labeled 231-Empty and 231-GATA3 cells as measured by flow cytometry. Bar graph shows % cells in S phase (mean +/− SEM).

Our microarray analysis of 231-Empty vs. 231-GATA3 cells also revealed that BMP5 was highly upregulated upon GATA3 overexpression. We confirmed by Q-RT-PCR a more than 200-fold increase in BMP5 expression in 231-GATA3 compared to 231-Empty cells. Although BMP2 and BMP4 were also altered upon GATA3 expression, the expression changes were minimal compared to BMP5 (Figure 7A). We observed no detectable levels of BMP7 in 231-Empty or 231-GATA3 cells by Q-RT-PCR (data not shown). The changes in BMP5 expression are associated with the levels of phosphorylated Smad1/5/8. These Smads are phosphorylated upon activation of the BMP pathway and basal phosphorylation of these Smads is higher in 231-GATA3 cells compared to 231-Empty cells (Figure 7B). Upon BMP5 treatment, there were elevated p-Smad1/5/8 levels in 231-GATA3 cells compared to 231-Empty cells even though total Smad1 levels were reduced in 231-GATA3 vs. 231-Empty cells (Figure 7B).

In order to investigate whether the GATA3-induced increase in BMP5 expression may contribute to the cytostatic effects of TGFß1 on MB-231 cells, we treated 231-Empty cells with either TGFß1 or BMP5 alone or in combination. Whereas treatment of 231-Empty cells grown under SR-conditions with either TGFß1 or BMP5 alone resulted in no change in % S-phase (19%, 23% and 20% in SR, TGFß1 and BMP5 alone, respectively), combination treatment of 231-Empty cells with TGFß1 and BMP5 resulted in reduced % S-phase to 14% (p<0.05) compared to SR-conditions (Figure 7C). Since 231-GATA3 cells express very elevated endogenous levels of BMP5, treatment with BMP5 in combination with TGFß1 did not change the % S-phase compared with treatment of TGFß1 alone (Figure 7C). Although combining BMP5 with TGFß1 resulted in an inhibition of proliferation of 231-Empty cells in response to TGFß1, knock down of BMP5 in combination of an inhibitory antibody for BMP5 in 231-GATA3 cells did not revert the GATA3 effect on TGFß1proliferative response (data not shown). It is possible that this treatment did not sufficiently inactivate the high endogenous levels of BMP5 in these cells or that in 231-GATA3 cells in addition to BMP5, GATA3 is promoting additional changes that results in the switch in TGFß response.

**Figure 8:**
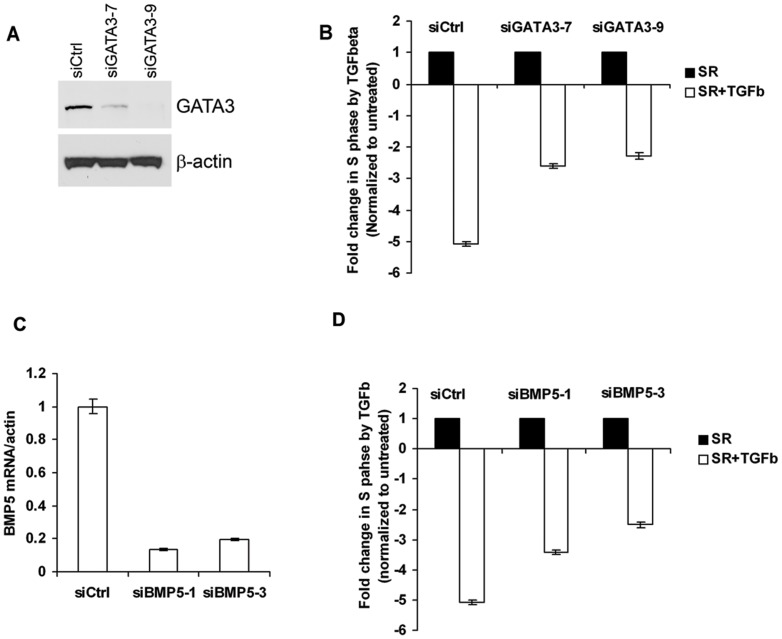
GATA3 and BMP5 knock down in MIII mammary epithelial cells decrease growth suppressive effect of TGFß. (A) Western blot analysis of GATA3 in MIII cells transfected with control or GATA-3 siRNA. (B) Flow cytometric analysis of MIII cells after transfection with control or GATA3 siRNA and TGFßtreatment. (C) Quantitative RT-PCR analysis of BMP5 expression after transfection with control or BMP5 siRNA. (D) Flow cytometric analysis of MIII cells after transfection with control or BMP5 siRNA and TGFßtreatment. More cells were in proliferative state (S-phase) after knockdown of GATA-3 (B) or BMP5 (D) and subsequent TGFß treatment than control siRNA-transfected cells treated with TGFß All values were normalized to that of the TGFß untreated cells.

To further explore the role of GATA3 and BMP5 in TGFß response without ectopic expression of GATA3, we used the MIII cell line. These cells are a tumorigenic derivative of the breast epithelial cell line MCF10A transformed with the activated H-ras oncogene [24]. Previously it has been shown that treatment with TGFß1 inhibits growth of MIII cells [25]. Furthermore, we detected a robust expression of GATA3 protein in these cells that was significantly decreased by siRNA targeting of GATA3 (Figure 8A). Similar to the response of 231-GATA3 cells to TGFß1, treatment of MIII cells with TGFß1 resulted in inhibition of cell proliferation in MIII cells transfected with control non-targeting siRNA. However, the growth suppressive effect of TGFß was blunted by two siRNAs against GATA3 (Figure 8B). The same cells express low, but detectable levels of BMP5 by quantitative RT-PCR. Knock down of BMP5 in MIII cells also partially diminished the inhibitory effect of TGFbeta on cell proliferation (Figure 8C and D), suggesting that BMP5, in fact, contributes to the inhibitory effect of TGFbeta on the proliferation of MIII cells.

## Discussion

The paradoxical effects of TGFßeither inducing or inhibiting proliferation have been extensively studied in several systems. However, the mechanisms that regulate these opposing responses to TGFß remain enigmatic. Early stage breast cancers are responsive to the cytotastic effects of TGFß, in contrast to late stage breast cancers that continue to proliferate in the presence of TGFß and acquire a more invasive phenotype [16]. The development of resistance to the cytostatic effect of TGFß may be a critical step during metastatic progression. In this study, we provide a new model to further investigate the switch in TGFß responsiveness in the well characterized triple-negative breast cancer cell line, MB-231. This cell line has been extensively investigated to ascertain effectors involved in metastasis and to investigate TGFß response [26], [27]. Our results demonstrate that ectopic expression of the differentiation transcription factor GATA3 in this well characterized metastatic cell line results in sensitization of the cells to the TGFß cytostatic response and promotion of MET, and propose that the expression of BMP5 contributes to this response. Our previous findings demonstrating that GATA3 expression in MB-231 cells induces a more luminal phenotype [5] and that proliferation of these cells is inhibited by TGFß are consistent with a previous report that demonstrated that the TGF pathway is less active in breast epithelial cells with a luminal phenotype [28].

Tumors from breast cancer patients that express markers of EMT are more commonly associated with the basal subtype [29] and with the claudin-low subtypes in breast cancer cells [30]. We have also previously shown that ectopic GATA3 expression in the basal triple-negative MB-231 cell line transdifferentiates these cells towards a more luminal subtype based on microarray profile analysis [5]. We now show that GATA3 not only transdifferentiates MB-231 cells towards a luminal subtype but that GATA3 also promotes MET in these cells. Therefore, the same mechanisms that direct cells towards the more aggressive basal subtype may be the same events that drive breast cancer cells towards EMT resulting in a more metastatic phenotype. Thus, transcription factors driving subtype specification may also regulate EMT/MET and metastatic potential in breast cancers. Furthermore, acquisition of an EMT phenotype has been associated with the acquisition of stem cell phenotype [11]. Further studies will decipher the interplay existing between these major pathways.

One potential mechanism utilized by GATA3 to promote all these changes is through epigenetic changes. We observed a reduction in methylation at the E-cadherin promoter and by methylation array we previously observed global changes in methylation (data not shown). Therefore, it is plausible that the epigenetic modifications promoted by GATA3 may result in the hypermethylation of transcription factors or genes associated with EMT.

Several mechanisms have been proposed to describe the paradoxical role of the TGFß proliferative response. One known mechanism is through modulation of disabled homolog 2 (DAB2) protein expression levels. DAB2 is an adaptor protein that regulates clathrin-mediated endocytosis [31]. Small cell carcinoma (SCC) cell lines expressing elevated levels of DAB2 remain sensitive to the anti-proliferative effects of TGFß. In contrast, cells with reduced expression of DAB2 continue to proliferate upon TGFß stimulation [31]. Furthermore, cells become resistant to the TGFß cytostatic response upon DAB2 knock down [31]. In addition to DAB2, the transcription factor C/EBPßa member of the basic leucine zipper family of transcription factors, has been implicated in altering the response to TGFß [32]. C/EBPß exists as 3 different isoforms, LAP1, LAP2 and LIP. LIP has a dominant negative effect on LAP1 and 2 that function as transcriptional regulators. Breast cancer cells with an elevated LIP∶LAP ratio were shown to escape the TGFß cytostatic response that correlates with a poorly differentiated phenotype and poor prognosis in breast cancer patients [32]. GATA3 was shown to interact with C/EBPα and ß in adipocytes and abrogate adipocyte differentiation [33]. Thus, GATA3 may also influence the TGFß response in breast cancer cells by influencing the response through C/EBP transcription factors. Further studies will be needed to more precisely decipher potential novel mechanisms of GATA3 interactions with the TGFß signaling pathways.

In addition to activating Smads, TGFß treatment can also activate non-canonical pathways. TGFß treatment of human mammary epithelial cells (HMECS) or normal murine mammary gland (NMUMG) cells activates MAPK [34], [35]. In HMECs, TGFß stimulation alone was not sufficient to promote EMT. Co-administration of ionizing radiation with TGFß predisposes HMECs to undergo TGFß dependent EMT through activation of MAPK [34]. Furthermore, inhibition of MAPK with UO126 abrogated TGFß-mediated EMT [34], [35]. Interestingly, we observed reduced basal activation of MAPK in 231-GATA3 vs. 231-Empty cells. Therefore, the failure of TGFß to promote EMT in 231-GATA3 cells could be due in part to the failure to reach a minimal thereshold of activation of the MAPK pathway upon TGFß stimulation. Although activation of Akt was also implicated in the TGFß-mediated EMT response in NMUMG cells [36], 231-GATA3 cells did not show changes in Akt phosphorylation compared to 231-Empty cells (data not shown).

Although, the role of TGFß signaling in cancer and EMT has been extensively studied, there is increasing evidence that other members of the TGFß superfamily, BMPs, also play an important role in cancer progression. Activation of BMP signaling results in phosphorylation of Smad1/5/8 which competes with Smad2/3 for binding to the co-Smad4. BMP7 decreased cell growth in HCC1954, MDA-MB-361, T-47D and ZR-75-30 cells [37]. BMP9 and BMP2 inhibited proliferation in MB-231 cells [38], [39]. BMP7 treatment reduced tumorigenesis in MB-231 xenografts [40] and reversed the TGFß-induced EMT in renal tubular epithelial cells [41]. BMP5 can reduce proliferation in PANC-1 cells [42] but the role of BMP5 in breast cancer is unknown.

The crosstalk between TGFß and BMP5 is beginning to be deciphered. BMP5 has been shown to reverse the TGFß induced EMT and cell transformation of human kidney cells with a significant downregulation in α-smooth muscle actin expression and a significant increase of ZO-1, a marker of MET [43]. More recently, the repression of BMP5 levels by TGFß1 has been directly implicated in the induction of EMT, with BMP5 acting as a suppressor of TGFß-mediated Snail expression and downregulation of E-cadherin [44]. These reports are in accordance with our observations of increased E-cadherin and ZO-1 levels and reduced Snail-1 levels in 231 cells overexpressing GATA-3. The significant upregulation of BMP5 in 231-GATA3 cells may dampen the response of 231 cells to TGFbeta by enforcing a more epithelial phenotype through the expression of epithelial markers and reducing the expression of EMT markers.

Here we have shown for the first time that although BMP5 or TGFß alone has little effect on proliferation in MB-231 cells, combined treatment of BMP5 with TGFß has a cytostatic effect on MB-231 cells. Additionally, we demonstrated that the inhibitory effect of TGFß on the proliferation of MIII cells is suppressed if GATA3 or BMP are knocked-down by siRNA, further demonstrating an interaction between these genes and the TGFß signaling pathway. This suggests a potential additional mechanism contributing to the switch in the TGFßresponseß

An interplay between GATA3 and TGFß is also evident during T cell differentiation. Both TGFß and GATA3 play important roles in modulating the T Helper (Th) lineage. GATA3 drives Th cells into Th2 differentiation and suppresses Th1 specification [1]. GATA3 directly activates expression of Th2 specific cytokines, IL-4, IL-5 and IL-13 and suppresses Th1 specific cytokines, IFN-γ and IL-12 [1]. In contrast, TGFß inhibits Th2 development through inhibition of GATA3 expression [45]. Furthermore, GATA3 abrogates TGFß dependent FOXP3 induction, a factor expressed in naïve T cells resulting in the inhibition of the development of iTreg cells [46]. The results of this study demonstrate for the first time that an interplay between TGFß signaling and GATA3 also exists in breast cancer. Further studies may unravel a potential direct link between GATA3 and TGFß signaling during mammary gland development and luminal/basal differentiation.

Given the important role of GATA3 in subtype specification and the TGFß dependent EMT phenotypic transition, it is plausible that GATA3 may also be critical in mediating responses to therapy. Breast cancer patients receiving the aromatase inhibitor, letrozole, showed enrichment in claudin-low patterns post-treatment compared to pretreated tumors. Further analysis of mesenchymal markers by immunofluorescence and automated quantitative analysis (AQUA) revealed an enrichment of mesenchymal markers in 26 paired patient samples post-treatment [47]. These findings suggest that tumors that have undergone EMT or express a mesenchymal phenotype may be more resistant to current therapeutic approaches [47]. Since GATA3 is associated with a more epithelial and luminal subtype, an increased understanding of the mechanisms utilized by GATA3 to reverse EMT and TGFß response and to promote a more luminal subtype may also lead to the identification of novel approaches to sensitize resistant breast cancers to therapies. Therefore, the 231-GATA3 and 231-Empty cell line model we have developed may not only serve to further elucidate the underlying mechanisms controlling the TGFß proliferative response, but it may also serve as a good model to investigate therapeutic response in luminal vs. basal breast cancer cells.
